# Mitochondrial-targeted therapeutics in oral squamous cell carcinoma: molecular and therapeutic implications

**DOI:** 10.3389/fmed.2026.1780863

**Published:** 2026-05-11

**Authors:** Saichao Zhou, Liye An

**Affiliations:** Department of Pediatrics, Preventive Dentistry and Orthodontics, Institute of Dentistry, I.M. Sechenov First Moscow State Medical University (Sechenov University), Moscow, Russia

**Keywords:** apoptosis, bruxism, mitochondrial, oral squamous cell carcinoma, ROS, targeted therapeutics

## Abstract

Oral squamous cell carcinoma (OSCC) is an aggressive malignancy characterized by frequent resistance to conventional chemoradiation, a phenotype increasingly linked to mitochondrial dysregulation. Accumulating evidence suggests that mtDNA mutational burden/signatures and broader metabolic reprograming may contribute to this resistant phenotype, but their predictive value and causal roles remain incompletely defined. Unlike prior syntheses that primarily catalog downstream phenotypic outcomes such as apoptosis or generalized oxidative stress, this comprehensive narrative review adopts a mechanism-driven framework centered on organelle-specific vulnerabilities and translationally relevant delivery strategies. We evaluate candidate therapeutics targeting electron transport chain (ETC) function, the mitochondrial apoptotic machinery, and redox homeostasis, while distinguishing OSCC-specific evidence from broader head and neck or pan-solid-tumor data. We further examine key barriers to clinical implementation, including intratumoral heterogeneity, limited tumor-selective mitochondrial delivery, and the lack of validated predictive and pharmacodynamic biomarkers. Overall, this review provides a cautious translational roadmap for testing mitochondria-directed strategies in OSCC and highlights priorities for biomarker-enriched, mechanism-informed clinical development.

## 1 Introduction

OSCC is one of the most prevalent malignancies arising in the mucosal linings of the oral cavity, including the tongue, mouth floor, and buccal mucosa. OSCC accounts for over 40% of head and neck cancers and represents a major cause of cancer-related mortality worldwide (1). The development of OSCC is associated with various risk factors such as tobacco smoking, excessive alcohol consumption, and poor oral hygiene (2). In addition, chronic mechanical irritation, including parafunctional habits such as bruxism, has been proposed as a potential cofactor that may contribute to mucosal injury and local inflammatory microenvironments, although its direct causal role in OSCC remains incompletely defined.

### 1.1 The Pathophysiological convergence of oncogenic drivers on mitochondrial dysfunction

At the molecular level, the carcinogenesis of OSCC is driven by a complex interplay of genetic and epigenetic alterations. However, rather than acting in isolation, canonical oncogenic drivers such as TP53 mutations, EGFR amplification, and specific metabolic alterations functionally converge to dysregulate core mitochondrial processes, including apoptotic priming, metabolic flux partitioning, and redox buffering capacity (3–5).

The tumor suppressor gene TP53 is frequently altered in OSCC/HNSCC. Beyond the loss of canonical transcriptional activity, p53 is also functionally linked to mitochondrial apoptosis and metabolic regulation. Recent mechanistic reviews indicate that wild-type p53 can translocate to mitochondria and interact with Bcl-2 family proteins at the outer mitochondrial membrane, thereby facilitating mitochondrial apoptotic signaling and MOMP, whereas loss of wild-type p53 function may raise the apoptotic threshold (6). In parallel, p53-dependent regulation of SCO2 has been implicated in the maintenance of cytochrome c oxidase assembly and oxidative phosphorylation, suggesting that TP53 disruption may contribute to the glycolytic shift observed in head and neck squamous cell carcinoma (4, 7, 8). Accordingly, in OSCC, TP53 alteration is more appropriately interpreted as a contributor to mitochondrial apoptotic dysfunction and metabolic reprograming, rather than as a single, fully resolved mitochondrial mechanism.

Similarly, aberrant EGFR signaling, which is common in OSCC, is closely associated with downstream PI3K/AKT activation and glycolytic reprograming. In the broader cancer literature, AKT signaling has been shown to promote mitochondrial association of hexokinase 2 (HK2), and mitochondria-bound HK2 can interact with VDAC to stabilize mitochondrial function and suppress apoptosis. In OSCC, recent evidence supports a role for HK2 in promoting glycolytic dependence and malignant progression, but the precise EGFR→ AKT→ HK2→ VDAC→ Bax axis should still be framed as a biologically plausible mechanistic model rather than a fully established OSCC-specific pathway (5, 9–14). Therefore, this signaling network is best described as linking growth-factor signaling to mitochondrial metabolic coupling and apoptotic resistance in OSCC, while still requiring more direct mechanistic validation in this disease context.

Beyond gross genomic alterations, mitochondrial homeostasis in OSCC may also be influenced by dysregulation of SIRT3, a major mitochondrial NAD + -dependent deacetylase. In OSCC-related literature, reduced SIRT3 deacetylase efficiency has been reported in association with a mutation near the catalytic region, and disruption of the miR-31–SIRT3 axis has also been shown to impair mitochondrial activity and increase oxidative stress; these findings have been reiterated in recent oral-cancer-focused reviews (15–17). More generally, recent reviews identify SOD2 and IDH2 among the key SIRT3-regulated antioxidant and metabolic targets involved in mitochondrial redox homeostasis (18, 19). For this reason, in the OSCC setting it is more accurate to state that SIRT3 dysfunction may weaken mitochondrial redox buffering and favor oxidative stress-associated tumor progression, rather than to imply that a uniform class of “SIRT3 SNPs” has already been established as a common mechanistic driver across OSCC.

### 1.2 Mitochondrial DNA (mtDNA) mutations as determinants of therapeutic vulnerability

In addition to nuclear genomic lesions, somatic mitochondrial DNA (mtDNA) alterations are recurrent in OSCC, although their reported prevalence varies substantially across cohorts rather than converging on a single uniform estimate. Earlier studies identified tumor-associated mtDNA mutations in 77.8% of oral cancers of betel-quid chewers and in 49% of primary head and neck squamous cell carcinomas, whereas more recent deep-sequencing studies in OSCC have demonstrated increased heteroplasmy levels and enrichment of potentially functional protein-coding variants in tumor tissue relative to matched benign mucosa (20–23).

Crucially, these mtDNA alterations should not be viewed merely as passive passenger events. In gingivobuccal OSCC, somatic mtDNA mutations have been shown to include positively selected non-synonymous variants, and mutations affecting mitochondrial respiratory genes were associated with an increased risk of lymph-node metastasis, reduced mtDNA copy number, and lower expression of mitochondrial transcripts, collectively supporting a potential impact on ETC integrity, mitochondrial respiration, and redox homeostasis (24). Likewise, prospective OSCC sequencing data demonstrated significantly higher heteroplasmy levels in tumor samples than in matched benign tissue, with coding-region mtDNA mutations showing associations with worse survival, further suggesting that at least a subset of mtDNA lesions may shape clinically relevant tumor phenotypes rather than merely accumulate neutrally (22).

On this basis, it is reasonable to hypothesize that OSCC tumors harboring a higher burden of functionally relevant mtDNA alterations may display distinct redox states and metabolic dependencies. This interpretation is also consistent with experimental work showing that mtDNA alterations may influence cisplatin responsiveness in OSCC models, as well as with broader cancer literature proposing heteroplasmy shifting as a determinant of tumor progression and treatment response (25, 26). Accordingly, integrated profiling of mtDNA mutational patterns, heteroplasmy, and redox-associated phenotypes may help identify biomarker-defined subsets of OSCC for future mitochondria-directed therapeutic strategies, risk assessment, and tumor surveillance (23, 27).

Beyond bioenergetics, mitochondria regulate cell fate through modulating apoptotic signaling cascades. Mitochondrial outer membrane permeabilization liberates pro-apoptotic factors like cytochrome c to activate caspase-dependent programed cell death. Defective mitochondrial apoptosis enables evasion of cell death, promoting cancer cell survival (28). Additionally, dysfunctional mitochondria are a major source of ROS which can damage DNA and drive mutagenesis and tumorigenesis when antioxidant mechanisms are overwhelmed (29).

Mitochondrial-targeted therapies represent an emerging paradigm for precision cancer treatment that aims to exploit the integral roles of mitochondria in tumor cell metabolism, survival, and death (30). Current strategies center on developing small molecules, peptides, and nanocarriers that selectively accumulate in mitochondria to modulate key signaling pathways (31). Recent studies reveal multifaceted links between mitochondrial biology and therapeutic efficacy as well as resistance mechanisms in OSCC. For instance, natural compounds like NRC-03 can induce OSCC cell apoptosis by activating the mitochondrial apoptotic pathway, including cytochrome c release and caspase activation. Mitochondrial ROS production can also trigger mPTP opening and cytochrome c release to drive OSCC cell death (32, 33). Additionally, increased mitochondrial fission can elevate ROS and cytochrome c levels, enhancing apoptosis (34). However, other mitochondrial properties may enable OSCC cells to evade therapies. Mitophagy can degrade damaged ROS-producing mitochondria, attenuating oxidative stress-induced cytotoxicity (35). Further, mitochondrial DNA mutations alter metabolic pathways for continued ATP production to support OSCC cell survival despite chemoradiation treatment (32). Components of the tumor microenvironment like cancer-associated fibroblasts can also maintain OSCC mitochondrial function and antioxidant capacity, mediating resistance (22, 36).

In summary, mitochondrial-targeted therapies offer promise but require more rigorous evaluation to fully establish their future utility as part of the oncological armamentarium. Continued development of this unique class of precision therapeutics may provide clinical benefit for patients with certain mitochondrial-dependent cancers.

### 1.3 Search strategy and selection criteria

To improve methodological transparency, this article is explicitly presented as a comprehensive narrative review aimed at providing a mechanism-oriented and translational synthesis of the literature, rather than a systematic review or meta-analysis. Accordingly, although a PRISMA flow diagram was not applied, the literature search and selection process was structured to ensure transparency and thematic consistency.

A literature search was conducted in PubMed/MEDLINE, Web of Science, and Scopus for studies published up to March 2026. The search strategy combined the following keywords and Medical Subject Headings (MeSH) terms: (“oral squamous cell carcinoma” OR “OSCC” OR “head and neck squamous cell carcinoma”) AND (“mitochondria” OR “mitochondrial dysfunction” OR “oxidative phosphorylation” OR “reactive oxygen species”) AND (“targeted therapy” OR “apoptosis” OR “gene therapy” OR “nanoparticles”).

To define the scope of this review more clearly, the following eligibility criteria were applied.

Inclusion criteria: Peer-reviewed original research articles and selected high-quality review articles relevant to the topic. Studies involving *in vitro* mechanistic experiments, *in vivo* preclinical models, or clinical trials related to mitochondrial dysfunction or mitochondria-targeted therapy in OSCC or, when directly relevant, broader HNSCC. Articles published in English. Studies published primarily between January 2016 and March 2026, with particular attention to recent advances from 2022 to 2026; earlier highly foundational studies were included only when necessary for conceptual context. Studies with direct thematic relevance to mtDNA alterations, mitochondrial metabolism, redox regulation, apoptotic signaling, mitophagy/dynamics, or mitochondria-targeted therapeutic interventions.

Exclusion criteria: Non-English publications. Unverified preprints. Conference abstracts without accessible full texts. Studies lacking peer review. Articles with insufficient mechanistic relevance to mitochondrial biology in OSCC/HNSCC. Purely descriptive studies that did not provide meaningful mechanistic, translational, or therapeutic insight. Studies with major methodological limitations judged likely to compromise interpretability, including inadequate biological replication or insufficient statistical support for the conclusions presented. Because this review is narrative rather than quantitative, strict numerical sample-size thresholds were not used as absolute exclusion criteria. Instead, studies were evaluated according to mechanistic rigor, methodological clarity, and translational relevance. This approach was intended to balance breadth of coverage with critical selectivity.

## 2 Molecular mechanisms of mitochondrial dysfunction in OSCC

Mitochondria are not merely passive energy factories; they are dynamic signaling hubs that actively dictate cell fate. In OSCC, the dysregulation of key metabolic enzymes and tumor microenvironment (TME) crosstalk fundamentally rewires mitochondrial homeostasis, driving disease progression and therapy resistance. To move beyond descriptive associations, it is critical to dissect these phenomena by analyzing their physiological roles, their specific dysregulation in OSCC, the resulting malignant phenotypes, and the preclinically validated therapeutic strategies designed to exploit them.

### 2.1 The PKM2 axis: hijacking mitochondrial pyruvate flux

(a) Physiological Role: Pyruvate kinase muscle isozyme M2 (PKM2) catalyzes the final step of glycolysis and is an important regulator of glycolytic flux. Functionally, its activity state helps determine whether glucose-derived carbon is efficiently converted to pyruvate for mitochondrial oxidation or retained within aerobic glycolytic and biosynthetic programs.
(b) Dysregulation in OSCC: In OSCC, the PKM2/PKM1 ratio is markedly elevated compared with adjacent normal mucosa and oral epithelial dysplasia, and PKM2 expression is significantly associated with tumor progression (37). Furthermore, PKM2 can undergo aberrant nuclear translocation in OSCC cells, particularly under EMT-promoting conditions (38, 39).
(c) Malignant Phenotype: This functional redistribution lowers effective pyruvate kinase activity and favors aerobic glycolysis over mitochondrial pyruvate oxidation, thereby reinforcing the Warburg phenotype. Simultaneously, nuclear PKM2 promotes malignant progression through non-metabolic transcriptional programs: in OSCC it has been shown to induce EMT through post-translational repression of TGIF2, and to enhance invasion through ETS-1-dependent upregulation of MMP-9 (38, 39).
(d) Therapeutic Strategies: This dual metabolic-transcriptional dysregulation identifies PKM2 as a potential mitochondria-associated therapeutic vulnerability. However, in OSCC this strategy is more appropriately framed as a preclinical rationale than as a fully validated disease-specific intervention, because the directly available evidence more clearly supports anti-tumor effects from PKM2 inhibition or depletion, including reduced proliferation/invasion and induction of apoptosis/autophagy (37, 40).

### 2.2 CAF-derived lactate supporting OXPHOS via intracellular NADH generation

(a) Physiological Role: Nicotinamide adenine dinucleotide (NADH) is a principal electron donor for mitochondrial Complex I, where its oxidation contributes to electron transfer and proton pumping during oxidative phosphorylation (41, 42).
(b) Dysregulation in OSCC: While OSCC cells often display enhanced glycolysis, they also retain substantial metabolic plasticity and can exploit stromal support from the tumor microenvironment. In a CAF subset characterized by high ITGB2 expression, activation of the PI3K/AKT/mTOR axis promotes glycolytic reprograming and increased lactate production. This stromal metabolic output can then be reutilized by adjacent OSCC cells to support mitochondrial energy metabolism (43, 44).
(c) Malignant Phenotype: Importantly, the available evidence does not indicate that OSCC cells directly import extracellular NADH from CAFs. Rather, CAF-derived lactate is taken up by OSCC cells, in part through MCT1-dependent mechanisms, and metabolized to generate intracellular reducing equivalents, including NADH, which can then support mitochondrial oxidative phosphorylation and ATP production (43). This form of stromal–tumor metabolic coupling is therefore better described as a reverse Warburg-like interaction that enhances bioenergetic flexibility, sustains proliferation under nutrient stress, and may reduce dependence on glycolysis alone (45, 46).
(d) Therapeutic Strategies: From a therapeutic perspective, this pathway is more appropriately targeted at the level of CAF metabolic reprograming, lactate transport/utilization, or tumor-cell OXPHOS dependence, rather than being framed as blockade of “direct NADH import.” In the original OSCC study, inhibition of mitochondrial OXPHOS with metformin attenuated the proliferative effect of ITGB2-high CAF-conditioned medium, supporting the concept that stromal lactate-fueled mitochondrial metabolism represents a targetable vulnerability (47). Accordingly, potential intervention points include suppression of the ITGB2/PI3K/AKT/mTOR axis in CAFs, interference with lactate transport/metabolism, and selective disruption of mitochondrial respiration in metabolically supported OSCC cells (45, 48).

### 2.3 The Warburg effect as an evasion of mitochondrial apoptosis

(a) Physiological Role: Healthy oral epithelial cells rely substantially on mitochondrial pyruvate oxidation to sustain efficient ATP production and redox homeostasis, with pyruvate transport through the mitochondrial pyruvate carrier (MPC) and oxidation by the pyruvate dehydrogenase complex representing central control points of this process (49, 50).
(b) Dysregulation in OSCC: The classical Warburg effect—characterized by enhanced aerobic glycolysis and preferential conversion of pyruvate to lactate even under normoxic conditions—is a recognized metabolic feature of OSCC and is associated with tumor progression, metastasis, and therapeutic failure (10, 51). However, in the OSCC context, this shift is more accurately described as a relative redirection of pyruvate away from mitochondrial oxidation, with evidence for reduced PDH expression and upregulation of glycolysis-associated regulators, rather than as a fully established OSCC-specific mechanism of direct MPC suppression (12, 52).
(c) Malignant Phenotype: By diverting carbon flux away from mitochondrial oxidation, OSCC cells can support the accumulation of biosynthetic intermediates required for rapid proliferation while maintaining mitochondrial activity at a survival-compatible level. In functional terms, this metabolic state is associated with therapy resistance and may help constrain excessive mitochondrial ROS accumulation and intrinsic apoptotic signaling, rather than reflecting complete mitochondrial inactivity (10, 53, 54).
(d) Therapeutic Strategies: Reversing glycolytic dependence and re-engaging mitochondrial metabolism remains an active area of preclinical investigation in OSCC. However, BX795 should be described more precisely as a phosphoinositide-dependent kinase-1 (PDPK1) inhibitor, not as a direct inhibitor of the pyruvate dehydrogenase kinase/PDH axis. In OSCC models, BX795 enhanced sensitivity to cisplatin and radiotherapy, and this effect was associated with suppression of PDPK1/CD47/Akt-mediated glycolytic signaling, together with reduced expression of glycolysis-related proteins such as LDHA, PFKP, and PDK3, increased apoptosis, and metabolic reprograming (12). Therefore, it is more accurate to state that BX795 partially counteracts glycolytic reprograming and promotes therapy sensitization, rather than claiming that it directly relieves PDH inhibition or forcibly restores mitochondrial pyruvate oxidation. Metformin-related OSCC data likewise support the idea that increasing PDH expression can accompany reduced proliferation, migration, and enhanced apoptosis under hypoxic conditions (52).

**FIGURE 1:**
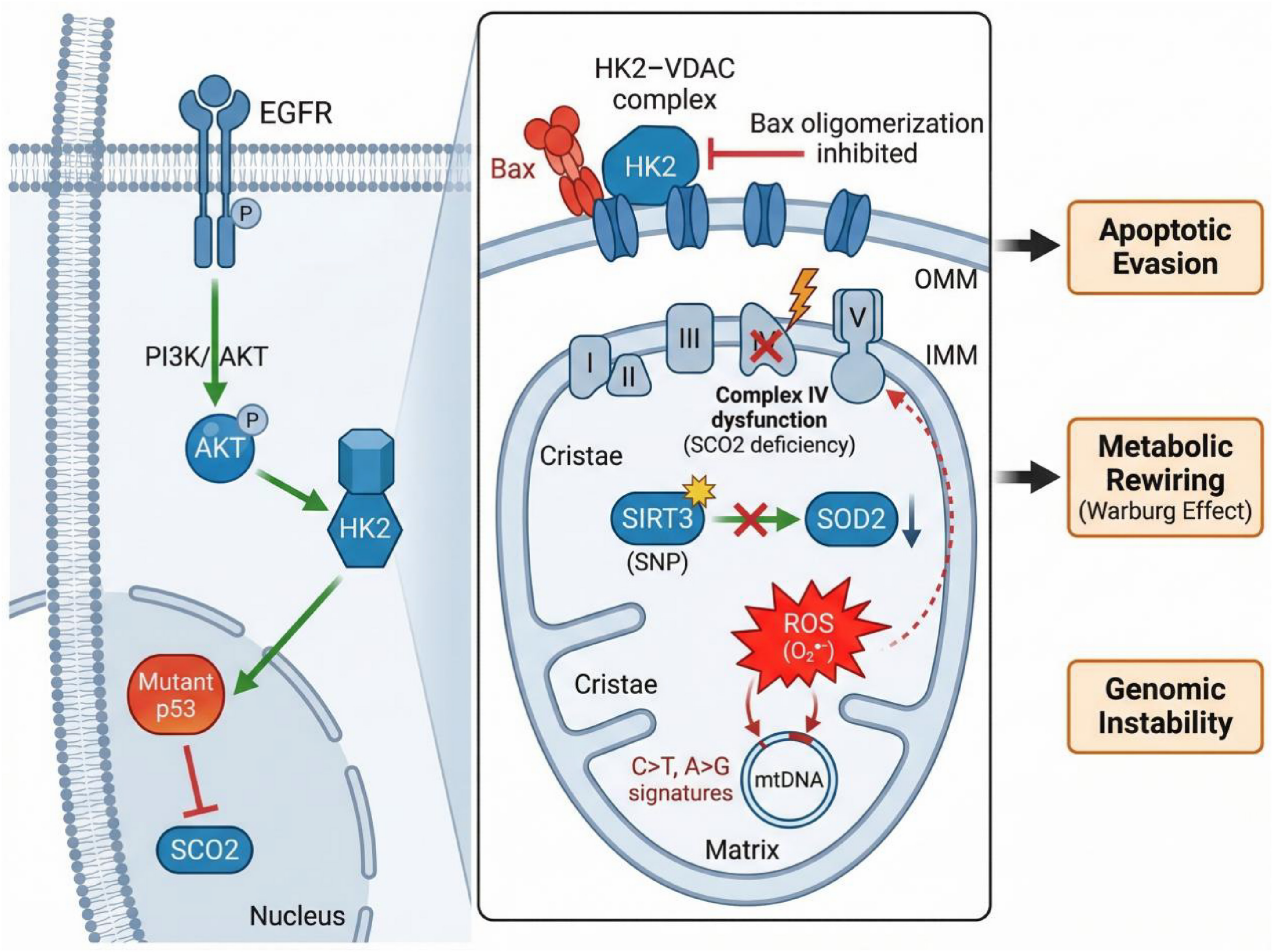
The pathophysiological convergence of oncogenic drivers on mitochondrial dysfunction in OSCC.

Canonical oncogenic signaling networks and genomic alterations may functionally interface with mitochondria to promote malignant phenotypes in OSCC (Figure 1). At the outer mitochondrial membrane (OMM), hyperactive EGFR/AKT signaling has been linked in broader cancer models to enhanced docking of hexokinase 2 (HK2) to the voltage-dependent anion channel (VDAC), favoring glycolytic flux and reduced apoptotic priming. Altered p53 signaling may also affect mitochondrial respiration, including through SCO2-dependent regulation of cytochrome c oxidase assembly, although direct OSCC-specific validation remains limited. Within the mitochondrial matrix, SIRT3 dysfunction or reduced SIRT3 activity may weaken antioxidant buffering through impaired deacetylation of enzymes such as SOD2, thereby favoring ROS accumulation. Persistent oxidative stress can damage mitochondrial DNA (mtDNA), and recurrent mtDNA alterations in OSCC have been proposed as candidate contributors to ETC dysfunction and therapeutic vulnerability rather than as universally validated drivers.

## 3 Mitochondrial-targeted therapeutic strategies for OSCC

Mitochondria are central regulators of bioenergetics, redox homeostasis, apoptotic priming, and organelle quality control in OSCC (55–57). Because these processes are frequently rewired during malignant progression, they provide multiple therapeutic entry points beyond conventional cytotoxic treatment (30, 31). In this section, mitochondria-targeted strategies are organized according to their major functional axes, including direct activation of the mitochondrial apoptotic machinery, disruption of oxidative metabolism and electron transport, modulation of mitochondrial dynamics and mitophagy, and mitochondria-directed drug delivery.

Rather than providing a purely descriptive list of compounds, the following discussion focuses on three translational questions: which mitochondrial process is being targeted, how such perturbation produces antitumor effects in OSCC models, and what major barriers may limit clinical application. These barriers mainly include metabolic plasticity, compensatory antioxidant responses, mitophagy-mediated escape, and the difficulty of achieving sufficient mitochondrial selectivity *in vivo* (30, 31). Taken together, these issues support a more critical and mechanism-based evaluation of mitochondrial targeting as a therapeutic strategy for OSCC.

**FIGURE 2:**
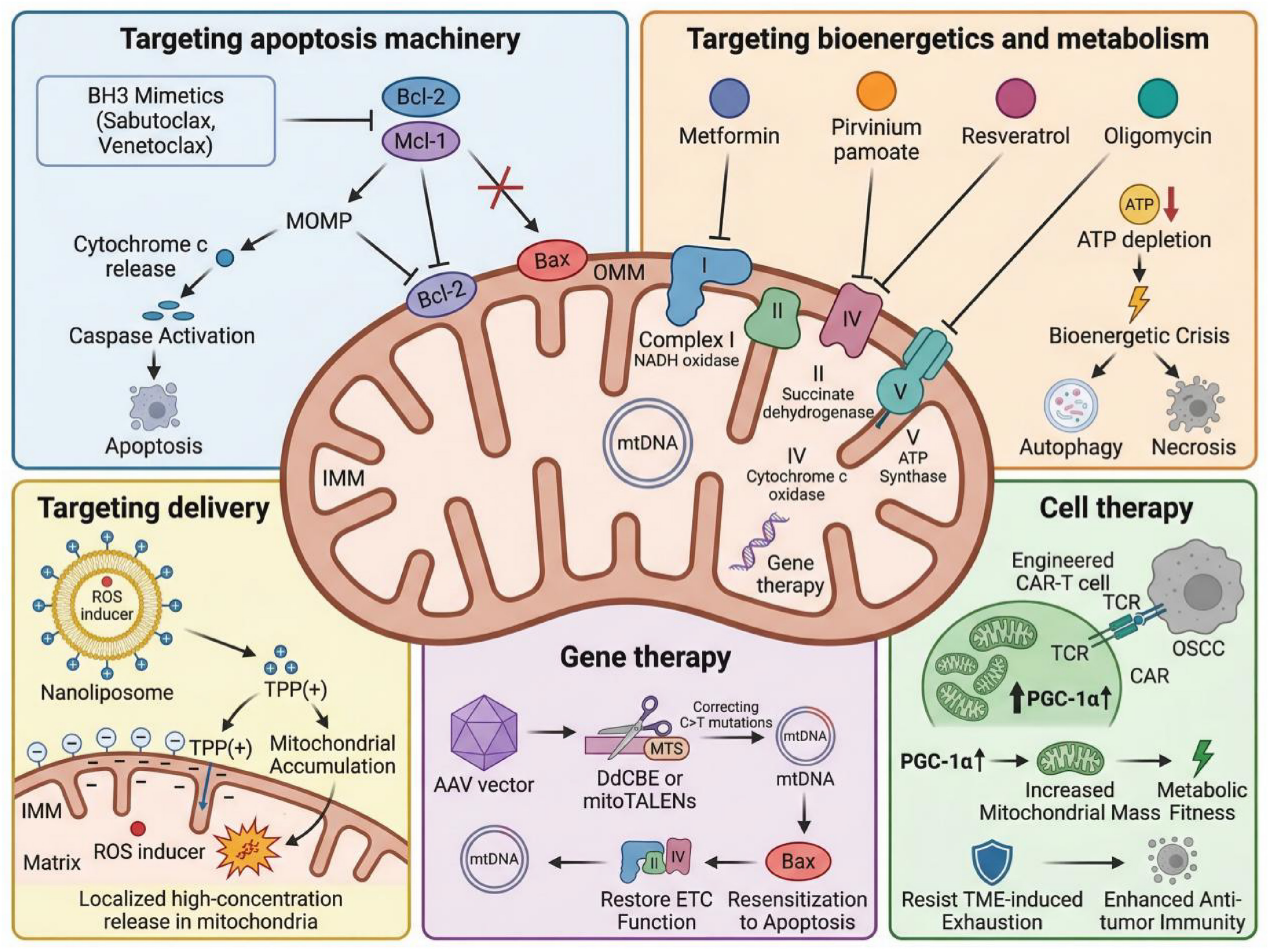
Mitochondrial-targeted therapeutic strategies in OSCC.

Mitochondria are central regulators of bioenergetics, redox homeostasis, apoptosis, and quality control in OSCC. As illustrated in Figure 2, current strategies include: (1) Targeting apoptosis machinery, where BH3 mimetics inhibit Bcl-2 family proteins to induce Bax/Bak-dependent MOMP, cytochrome c release, and caspase activation; (2) Targeting bioenergetics and metabolism, with agents disrupting the electron transport chain (ETC) and OXPHOS, leading to ATP depletion and ROS accumulation; (3) Targeted delivery systems, using TPP + -modified nanocarriers to enhance intramitochondrial drug accumulation and ROS generation; (4) Gene therapy, employing mitochondrial genome editing tools (e.g., DdCBE, mitoTALENs) to restore mitochondrial function; and (5) Cell therapy, enhancing mitochondrial fitness (e.g., via PGC-1α) to improve antitumor immunity.

### 3.1 Targeting the mitochondrial membrane and apoptotic machinery

Rather than merely observing apoptosis as a phenotypic outcome, a major therapeutic strategy involves directly engaging the molecular complexes that govern mitochondrial outer membrane permeabilization (MOMP). The Bcl-2 family of proteins serves as the primary gatekeeper of this process, presenting a highly druggable target axis in OSCC. Mitochondrial-targeted agents in preclinical OSCC models are compared in Table 1.

**TABLE 1:** Comparative analysis of representative mitochondrial-targeted agents in preclinical OSCC models.

Mechanistic class	Representative agents	Primary mitochondrial target	Key preclinical evidence in OSCC	Translational challenges and limitations
BH3 mimetics	Sabutoclax, Venetoclax	Anti-apoptotic Bcl-2 family proteins (Bcl-2, Mcl-1)	Directly induces MOMP and cytochrome c release; lowers the apoptotic threshold (58).	On-target toxicity (e.g., thrombocytopenia); rapid emergence of resistance via compensatory upregulation of untargeted anti-apoptotic members (e.g., Mcl-1).
ETC complex inhibitors	Metformin, Phenformin, Pirvinium pamoate	Complex I (Metformin) and Complex II (Pirvinium)	Restricts oxidative energy metabolism; diminishes tumor sphere formation in preclinical models (67).	Requires supraphysiological concentrations *in vivo*; risk of systemic lactic acidosis; tumors often adapt by hyper-activating alternative glycolytic pathways.
Pro-oxidant agents	NRC-03, Oxaliplatin	CypD-mPTP axis; ETC components	Triggers lethal mtROS burst, massive oxygen consumption, and PARP1-mediated parthanatos (32, 72).	Narrow therapeutic window due to collateral oxidative damage to normal tissues; robust resistance driven by Nrf2-mediated antioxidant compensation.
Metabolic reprograming modulators	BX795 (PDK1 inhibitor)	PDPK1/CD47/Akt-associated glycolytic signaling	Suppresses glycolysis-associated signaling, enhances cisplatin/radiosensitivity, and promotes apoptosis (12).	Indirect metabolic effects; pathway redundancy; no OSCC *in vivo* PD data; no responder biomarkers; no acquired-resistance models.
Mitochondrial kinase inhibitors	Machilin D, Ursolic Acid	PI3K/AKT/mTOR signaling axis to the mitochondria	Disrupts anti-apoptotic kinase signaling at the mitochondrial membrane, activating Bak/Bax (62, 64).	Broad kinase inhibition often leads to off-target toxicities and activates compensatory pro-survival kinase cascades (e.g., ERK upregulation).

Direct activation of pro-apoptotic Bcl-2 family proteins or inhibition of their anti-apoptotic counterparts can forcibly lower the apoptotic threshold in malignant cells. For instance, small molecule BH3 mimetics like Sabutoclax have demonstrated the ability to directly trigger MOMP and the subsequent release of cytochrome c (58). Similarly, targeting anti-apoptotic Bcl-2 proteins with agents such as venetoclax selectively displaces pro-apoptotic effectors, committing the cancer cell to programed death. Additionally, strategies that upregulate apoptosis regulatory factors, such as SMAC/DIABLO, via the targeting of inhibitors of apoptosis proteins (IAPs) using agents like the curcumin analog GO-Y078, further sensitize the mitochondrial membrane to permeabilization (59).

Beyond direct Bcl-2 targeting, several natural compounds alter membrane integrity by manipulating upstream kinase networks that physically interact with mitochondrial membrane components. The PI3K/AKT/mTOR and ERK axes directly phosphorylate Bcl-2 family members like Bim and Bad (60, 61). Compounds such as Machilin D (62), Xanol (63), and Ursolic acid (64) indirectly precipitate mitochondrial membrane collapse in OSCC by suppressing these oncogenic kinases, leading to the activation of Bak/Bax, loss of mitochondrial membrane potential, and caspase-3/9 activation (64).

### 3.2 Targeting mitochondrial metabolism and bioenergetics

Cancer cells, including OSCC, frequently exhibit metabolic plasticity. While the Warburg effect highlights an increased reliance on aerobic glycolysis, functional mitochondrial oxidative phosphorylation (OXPHOS) remains critical for tumor survival, macromolecule synthesis, and managing redox homeostasis (65). Consequently, targeting the Electron Transport Chain (ETC) and specific metabolic enzymes constitutes a formidable therapeutic approach.

Inhibitors of the mitochondrial respiratory chain directly suffocate the bioenergetic capacity of OSCC cells. Metformin and Phenformin, widely recognized as inhibitors of ETC complex I (NADH dehydrogenase), have shown significant potential in restricting OSCC energy metabolism (66). Furthermore, pirvinium pamoate functions as a complex II inhibitor, severely diminishing tumor sphere formation in various cancer cell lines (67). Other bioenergetic disruptors include resveratrol, which impairs Complex IV (68, 69), and oligomycin, a potent inhibitor of ATP synthase (Complex V) (70). Uncoupling agents like salinomycin further dismantle the proton motive force, uncoupling electron transport from ATP synthesis and inducing severe energetic crisis in OSCC (71).

Closely intertwined with mitochondrial bioenergetics is the generation and regulation of reactive oxygen species (ROS). While moderate ROS levels promote oncogenesis, excessive, unbuffered mitochondrial ROS (mtROS) triggers catastrophic macromolecular damage. Therefore, selectively amplifying mtROS to toxic levels by disrupting ETC electron flux is a validated strategy. For example, the natural compound NRC-03 forces massive oxygen consumption and lethal ROS release, compromising mitochondrial structural integrity and inhibiting OSCC xenograft growth (32). Oxaliplatin similarly induces PARP1-mediated parthanatos in OSCC by overwhelmingly augmenting ROS production and actively depleting antioxidant reserves like SOD and GSH (72).

### 3.3 Targeting mitochondrial dynamics, biogenesis, and quality control

Mitochondria are highly dynamic organelles that undergo continuous cycles of fission, fusion, and selective degradation to maintain function under metabolic and oxidative stress. In OSCC, dysregulation of these processes contributes to tumor survival and progression, but also exposes actionable vulnerabilities. Available evidence indicates that altered mitochondrial dynamics are directly involved in malignant phenotypes: mitochondrial ROS1 promotes mitochondrial fission, oxidative phosphorylation, ATP production, and invasion, whereas DRP1 inhibition induces mitochondrial elongation, suppresses stemness, and increases ferroptotic susceptibility (33, 53, 73).

A major conceptual challenge in this field is that mitophagy should not be described as uniformly tumor-promoting or uniformly tumor-suppressive in OSCC. Instead, the available evidence supports a context-dependent model. At the conceptual level, mitochondrial quality control can protect cells by clearing damaged mitochondria and limiting excessive oxidative stress, yet under other conditions the same process may support tumor adaptation and therapeutic escape (53).

In OSCC specifically, the direct evidence is bidirectional. On the one hand, excessive PINK1/Parkin-mediated mitophagy has been shown to accompany mitochondrial damage, ROS accumulation, and apoptosis in CAL-27 cells exposed to zinc oxide nanoparticles, indicating that overactivation of mitophagy can become cytotoxic (74). On the other hand, a recent recurrent/resistant OSCC study showed that PSMA2 contributes to chemo- and radioresistance by modulating mitochondrial dysfunction and mitophagy, and that a mitophagy inducer exerted antitumor effects in a PSMA2-overexpressing xenograft model (75). These findings indicate that mitophagy in OSCC is better understood as a bidirectional regulator of therapeutic response rather than a monolithic resistance mechanism.

Accordingly, therapeutic strategies targeting mitochondrial quality control in OSCC should be highly contextualized. In some settings, blocking cytoprotective autophagy/quality-control responses may enhance antitumor efficacy; for example, metformin induces autophagy in OSCC cells, and hydroxychloroquine enhances metformin-induced apoptosis and tumor suppression (76). In other settings, forcing excessive mitochondrial clearance may itself become therapeutically beneficial, as suggested by ZnO nanoparticle-induced PINK1/Parkin-mediated mitophagy and by the mitophagy-inducer response observed in PSMA2-associated resistant OSCC models (74, 75). Therefore, rather than treating mitophagy as a single-direction target, a more accurate framework is to modulate mitochondrial quality control according to tumor state, treatment context, and the dominant form of mitochondrial stress adaptation (53).

### 3.4 Mitochondria-targeted delivery strategies

A major impediment to realizing the clinical potential of the aforementioned targets is the difficulty of achieving sufficient intracellular drug concentrations within mitochondria, which are protected by a double-membrane architecture and a strong negative inner membrane potential (44).

To overcome these barriers, precision delivery strategies are actively being developed. Conjugation of therapeutic payloads to lipophilic cations such as triphenylphosphonium (TPP) exploits mitochondrial membrane potential to promote selective intramitochondrial accumulation of bioactive molecules in cancer cells (77). Moreover, mitochondria-targeted liposomes engineered with TPP-functionalized surfaces have demonstrated mitochondrial localization, pH-responsive drug release, increased ROS generation, and enhanced cytotoxicity in cancer models, thereby supporting the feasibility of precision subcellular delivery (78).

In OSCC specifically, advanced nanoplatforms such as Ru(II)-modified TiO2@siRNA nanoparticles further illustrate the therapeutic value of intracellularly targeted delivery. However, this system should be described more precisely as a hypoxia-adaptive photo-immunotherapeutic nanosystem rather than as a definitively mitochondria-targeted carrier, because the published evidence emphasizes lysosomal damage, HIF-1α silencing, and remodeling of the immune microenvironment (79). Therefore, these delivery approaches are best framed as strategies that improve intracellular precision, enhance tumor-selective mitochondrial stress, and may reduce off-target toxicity relative to untargeted mitochondrial disruptors, although further OSCC-specific validation remains necessary for true mitochondria-directed delivery platforms (46, 53).

### 3.5 The interplay of apoptosis and oxidative stress: mechanistic integration and critical evaluation

While the aforementioned mitochondrial-targeted agents act on distinct molecular sub-compartments, their ultimate cytotoxic effects in OSCC converge heavily on the dynamic interplay between reactive oxygen species (ROS) generation and the intrinsic apoptotic cascade. Rather than operating in isolation, these two pathways form a tightly coupled, self-amplifying network. Primary disruption of the electron transport chain (ETC) elevates mitochondrial ROS (mtROS), and this localized oxidative stress can promote cardiolipin oxidation, mitochondrial depolarization, and permeability transition. In turn, these events facilitate cytochrome c mobilization and caspase activation, particularly when coordinated with BAX/BAK-dependent outer membrane permeabilization (80–83). Conversely, primary engagement of the apoptotic machinery, including BH3 mimetic–driven mitochondrial apoptosis, activates BAX/BAK at the outer mitochondrial membrane and can further aggravate mitochondrial dysfunction and ROS accumulation, thereby reinforcing cell death signaling rather than acting as an entirely separate pathway (82, 83).

Despite the robust preclinical promise of mitochondrial-targeted strategies, major translational hurdles remain. First, the ubiquitous requirement for mitochondrial integrity in normal tissues raises the risk of on-target, off-tumor toxicity. This concern is particularly relevant for highly oxidative tissues such as the myocardium and the nervous system, where mitochondrial dysfunction and oxidative stress are tightly linked to tissue injury (46, 84). Second, OSCC cells display marked metabolic plasticity and adaptive resistance. Under mitochondrial stress, cancer cells can compensate by shifting toward glycolysis, and OSCC is now well recognized as a metabolically rewired tumor in which aerobic glycolysis contributes to progression, metastasis, and treatment failure (10, 85). Likewise, ROS-based therapies may be blunted by activation of the Nrf2/Keap1 antioxidant pathway. In OSCC, Nrf2 upregulation has been shown to promote radioresistance through enhancement of glycolysis and glutathione synthesis, supporting the view that antioxidant adaptation can buffer ROS-directed therapeutic injury (86). Therefore, comparing the mechanistic strengths and adaptive limitations of representative mitochondrial-targeted agents is essential for rational clinical translation in OSCC (53, 85). For metabolic reprograming modulators in particular, the most important unresolved preclinical gaps are the lack of OSCC *in vivo* pharmacodynamic confirmation that PDPK1/CD47/Akt signaling is durably suppressed in tumor tissue, the absence of flux-based evidence that mitochondrial pyruvate utilization is actually restored, and the lack of biomarker-defined responder criteria or well-characterized acquired-resistance models.

## 4 Rational design of mitochondrial-targeted combination therapies

While mitochondrial-targeted agents demonstrate potent single-agent efficacy in preclinical OSCC models, their clinical translation is frequently hindered by adaptive resistance and dose-limiting systemic toxicities. Consequently, the field is pivoting toward rationally designed combinatorial regimens. Moving beyond generic additive effects, a rigorous combination strategy must be grounded in precise mechanistic synergy, aiming to preempt resistance pathways and dismantle the tumor’s bioenergetic flexibility.

### 4.1 Mechanistic synergy with conventional chemotherapy

The most compelling rationale for combining mitochondrial-targeted agents with standard-of-care drugs such as cisplatin lies in their complementary cytotoxic mechanisms. Cisplatin primarily exerts antitumor activity by generating DNA adducts, thereby inducing DNA damage, cell-cycle arrest, and apoptosis. However, in OSCC, resistance commonly emerges through enhanced DNA damage repair and suppression of apoptosis, including upregulation of anti-apoptotic Bcl-2 family signaling (87, 88).

Co-administration of a mitochondrial bioenergetic modulator such as metformin may increase cisplatin responsiveness in OSCC, but this effect is more accurately attributed to metabolic stress signaling and apoptosis sensitization than to a fully established direct blockade of ATP-dependent DNA repair. In OSCC models, metformin enhanced cisplatin cytotoxicity through inhibition of the NF-κB/HIF-1α axis, and a more recent study further showed synergistic suppression of proliferation together with increased ROS, apoptosis, and AMPK activation (89, 90).

Likewise, combining cisplatin with apoptosis-sensitizing mitochondrial agents is mechanistically attractive because it lowers the threshold for mitochondrial cell death execution. In OSCC, the pan-BH3 mimetic obatoclax was shown to synergistically enhance cisplatin-induced apoptosis, an effect associated with degradation of the pro-survival protein Mcl-1 and increased conformational activation of Bak (91). In parallel, ROS augmentation has also been shown to increase cisplatin cytotoxicity in tongue squamous cell carcinoma cells by promoting apoptosis and autophagy, whereas ROS scavenging reduced these effects, supporting the idea that redox amplification can help convert cisplatin-induced stress into lethal mitochondrial signaling (92). Accordingly, the most accurate formulation is that mitochondrial-targeted combinations may help drive cisplatin-treated OSCC cells away from damage tolerance and toward irreversible mitochondrial apoptosis, rather than simply forcing a bioenergetic collapse of DNA repair alone (91, 92).

### 4.2 Preempting adaptive resistance mechanisms

As OSCC cells experience mitochondrial stress, they can engage compensatory survival programs, most notably autophagy/mitochondrial quality control and metabolic rewiring. In OSCC, metformin has been shown to induce protective autophagy, and hydroxychloroquine enhances metformin-induced apoptosis and tumor suppression, indicating that autophagic flux can buffer therapy-associated metabolic stress (76). However, mitophagy in OSCC should not be described as uniformly cytoprotective; rather, recent evidence indicates that it is context-dependent, as recurrent/resistant OSCC has been linked to PSMA2-associated modulation of mitophagy and mitochondrial dysfunction (75).

Likewise, OSCC exhibits substantial metabolic heterogeneity and plasticity, with oral cavity squamous cell carcinomas showing clinically relevant variation in coordinated oxidative phosphorylation and glycolysis programs (93). In broader preclinical cancer models, inhibition of OXPHOS alone can provoke compensatory glycolysis, whereas simultaneous blockade of OXPHOS and glycolysis can produce energy depletion and stronger antitumor effects (94, 95). In the OSCC setting, this principle is partially supported by a 4NQO-derived model in which combined targeting of autophagy and glycolytic metabolism with chloroquine and dichloroacetate improved control of tumor development and survival outcomes (96). Accordingly, dual-inhibition strategies remain mechanistically attractive in OSCC, but examples such as 2-deoxyglucose are more appropriately presented as translational rationale rather than as fully established OSCC-specific regimens.

### 4.3 The landscape of clinical translation in OSCC

Despite robust preclinical rationales, the clinical translation of mitochondrial-oriented therapies specifically tailored for OSCC remains in its early stages. In the oral cavity, human evidence is still dominated by short window-of-opportunity and chemoprevention studies rather than randomized therapeutic trials. For example, in non-diabetic patients with oral cavity squamous cell carcinoma, a prospective preoperative metformin study found that 10–14 days of treatment did not significantly support the hypothesis of improved tumor hypoxia–associated gene expression (97), whereas in oral premalignant lesions, a phase IIa metformin trial reported encouraging histologic responses and mTOR-pathway modulation, despite more modest clinical shrinkage (98).

There is therefore a conspicuous paucity of phase II/III trials dedicated specifically to OSCC. Within broader HNSCC, the clearest clinical experience still involves repurposed metabolic modulators such as metformin. A prospective phase I/II trial combining metformin with platinum-based chemoradiation in newly diagnosed HNSCC concluded that the regimen was tolerable, but that its radiosensitizing efficacy remained uncertain and would require randomized validation (99).

Apoptosis-targeting translation is also more limited than is sometimes implied. A 2022 overview of BH3 mimetics in HNSCC found that the literature was still dominated by preclinical studies and identified only one phase II human trial in that space (100). By contrast, the most clinically advanced apoptosis-sensitizing precedent in HNSCC has involved the IAP antagonist xevinapant, not a classical BH3 mimetic; notably, the phase III TrilynX study reported that adding xevinapant to platinum-based chemoradiotherapy did not improve event-free survival in unresected locally advanced HNSCC (101).

Bridging this translational gap will require OSCC-specific, biomarker-driven trials that separate oral-cavity disease from pooled HNSCC cohorts and match the relevant mitochondrial vulnerability to the most appropriate agent.

## 5 Emerging interfaces between mitochondrial targeting and gene/cell therapy

Recent advances in mitochondrial biology have created several conceptual interfaces with the broader fields of gene and cell therapy. In OSCC, however, these approaches should currently be viewed as emerging translational concepts rather than established therapeutic strategies, because the present evidence base is derived predominantly from mitochondrial genome-editing platform studies, mitochondrial disease models, and broader mitochondria-focused immunotherapy research rather than direct OSCC-specific therapeutic validation (102–104). Accordingly, this section highlights plausible future directions at the intersection of mitochondrial targeting and gene/cell therapy, while emphasizing the current need for direct OSCC-focused mechanistic and translational studies (22, 23). To avoid overinterpretation, the following subsections are framed as future-oriented translational interfaces supported mainly by non-OSCC platform data, not as therapeutic approaches already validated in OSCC.

### 5.1 Precision mitochondrial genome (mtDNA) editing

As discussed above, somatic mtDNA alterations and heteroplasmy are recurrent in OSCC/HNSCC and may contribute to biologically meaningful tumor heterogeneity (22, 23). Historically, direct engineering of the mitochondrial genome was considered technically difficult because conventional CRISPR/Cas systems are not readily applicable to mitochondria, in part owing to inefficient mitochondrial import of guide RNA; however, the development of mitochondria-targeted nucleases and base editors, particularly mitoTALENs and DddA-derived cytosine base editors (DdCBEs), has demonstrated that selective manipulation of mutant mtDNA and heteroplasmy is feasible in mammalian systems (102, 103, 105, 106).

In the context of OSCC, these tools should currently be regarded primarily as emerging research and early translational platforms rather than established therapeutic modalities. One plausible application would be to functionally interrogate recurrent mtDNA variants in patient-derived OSCC models, thereby clarifying whether specific lesions alter oxidative phosphorylation, ROS handling, or sensitivity to platinum-based therapy (22, 102). A second, more forward-looking possibility would be selective depletion of pathogenic mtDNA subclones or heteroplasmy shifting in premalignant or early neoplastic lesions, drawing on the broader proof-of-concept established in mitochondrial disease models (103, 105, 107). At present, no study has demonstrated therapeutic correction of a defined mtDNA lesion in OSCC *in vivo* using mitoTALENs, DdCBEs, or related mitochondrial editors; therefore, the immediate value of these tools in OSCC remains mechanistic interrogation, heteroplasmy modeling, and biomarker-oriented preclinical stratification.

Nevertheless, substantial barriers remain before such approaches can be considered therapeutically mature for OSCC, including inefficient tumor-restricted delivery, limited editing scope, uncertainty regarding heteroplasmy dynamics, and the risk of off-target editing. These limitations are especially relevant for DdCBE-based systems, for which off-target events and the need for further optimization have been explicitly highlighted in the current mitochondrial genome-editing literature (102, 103, 108).

### 5.2 Enhancing cellular immunotherapy via mitochondrial engineering

The metabolically hostile tumor microenvironment of OSCC, which is shaped by hypoxia, stromal crosstalk, and immune dysfunction, is likely to limit the efficacy of adoptive cellular immunotherapies such as CAR-T cells and tumor-infiltrating lymphocyte (TIL) transfer (46). More broadly, the solid-tumor microenvironment is characterized by nutrient deprivation, persistent antigen stimulation, hypoxia, and immunosuppressive signaling, all of which can drive mitochondrial insufficiency and T-cell dysfunction (104, 109).

A growing body of evidence indicates that improving mitochondrial fitness can enhance the performance of adoptively transferred T cells. In preclinical models, enforced PGC-1α expression promotes mitochondrial biogenesis, metabolic fitness, memory formation, and antitumor immunity in CD8 + T cells (110). In addition, an engineered Akt-resistant form of PGC-1α improved mitochondrial biogenesis, metabolic programing, and the *in vivo* efficacy of human anti-EGFR CAR-T cells in a solid-tumor model (111). However, these data derive from general solid-tumor or non-OSCC adoptive-cell-therapy models and therefore establish biological plausibility rather than OSCC-specific therapeutic efficacy.

Recent work has also shown that intercellular mitochondrial transfer can enhance mitochondrial respiration, spare respiratory capacity, tumor infiltration, and antitumor efficacy of CD8 + T cells in solid-tumor models, thereby supporting the concept of organelle-level metabolic reinforcement before adoptive transfer (112). This platform has subsequently been interpreted as a prototype for next-generation organelle-based cancer immunotherapy, but it remains a preclinical concept at present (113). Likewise, the mitochondrial-transfer platform has not yet been validated in an OSCC-directed adoptive-cell-therapy setting.

Accordingly, mitochondrial engineering may represent a plausible adjunctive strategy for future OSCC-directed cell therapy. However, direct therapeutic validation in OSCC remains very limited, and the current rationale is still extrapolated mainly from broader solid-tumor immunotherapy, T-cell metabolism, and mitochondrial-engineering studies rather than from OSCC-specific adoptive-cell-therapy trials (46, 104, 109). Important translational questions, including manufacturing compatibility, durability of metabolic reprograming, tumor specificity, and safety, therefore remain unresolved. Accordingly, in OSCC this concept should currently be interpreted as extrapolative and hypothesis-generating until disease-specific efficacy, trafficking, persistence, manufacturing compatibility, and safety data become available.

## 6 Rigorous preclinical toxicology evaluation and safety profiling

A robust and comprehensive toxicology evaluation in animal models constitutes an imperative step in the development of mitochondrial-targeted therapies. This evaluation is essential to thoroughly characterize the safety profile and to identify potential systemic toxicities of these novel therapeutic approaches prior to their translation into clinical trials involving human subjects. Mitochondria-targeted therapies can impact not only cancer cells but also normal, non-cancerous cells. This raises concerns about potential off-target effects, especially in tissues with high mitochondrial activity, such as muscle and nerve cells.

While initial preclinical studies in the context of OSCC have reported promising selectivity and cancer cell-specific toxicity, it is paramount to recognize that the mitochondria play critical roles in cellular function across all cell types (114). Thus, rigorous toxicology studies are warranted to comprehensively assess and exclude any potential risks of adverse effects on normal tissue function. These evaluations provide critical insights into any organ-specific toxicities and potential functional deficits. In addition to short-term experiments, long-term chronic toxicology testing over extended durations, spanning weeks to months, is equally imperative (115). Such long-term assessments are essential to identify any cumulative or delayed toxicities that may not become apparent in shorter studies. These chronic evaluations ensure a more comprehensive understanding of the safety profile of mitochondrial-targeted therapies over prolonged treatment periods. Complementary to animal studies, it is valuable to undertake toxicology screening in relevant human cell lines, three-dimensional organoid cultures, and induced pluripotent stem cell-derived tissues (116, 117). These *in vitro* systems can offer additional perspectives on potential mitochondrial liabilities and toxicities, further aiding in the early identification of safety concerns. However, it is essential to acknowledge the limitations of these *in vitro* models in replicating the complexities of intact organisms.

Venetoclax, a Bcl-2 inhibitor used in the treatment of chronic lymphocytic leukemia (CLL) and certain types of lymphomas, has demonstrated efficacy but may also lead to various toxicities and side effects. Common venetoclax-associated toxicities and approaches to mitigate them include tumor lysis syndrome (TLS), hematologic cytopenias, gastrointestinal distress, fatigue, and other side effects. TLS risk can be reduced through pre-treatment assessment and proactive management of electrolyte imbalances. Hematologic cytopenias necessitate regular blood count monitoring and intervention as needed. Gastrointestinal symptoms can be managed through dietary adjustments and medications, while fatigue management involves maintaining adequate rest and activity planning. Patients should promptly report any discomfort to their healthcare team. Tailoring the management plan for venetoclax treatment to individual patient needs and medical history is essential (118).

In summary, the extensive toxicology assessment in animal models stands as an indispensable next step in the development of mitochondrial therapeutics for OSCC and other cancer types. The knowledge generated from these preclinical safety studies will not only provide confidence in the translation of promising mitochondrial-targeted therapies into clinical trials but also guide the design of these trials to minimize risks and ensure vigilant monitoring for potential mitochondrial-mediated adverse effects. Ultimately, this rigorous preclinical evaluation is pivotal in the quest to enhance the outcomes and safety of mitochondrial-targeted therapies for the benefit of OSCC patients and beyond.

## 7 Conclusion and future directions

Although mitochondria-targeted strategies remain mechanistically attractive in oral squamous cell carcinoma (OSCC), their clinical translation is still at an early stage. As summarized in Table 2, only a limited number of mitochondria-acting agents have reached clinical evaluation in head and neck squamous cell carcinoma (HNSCC)/OSCC, and the currently available data support feasibility more strongly than definitive efficacy. For example, metformin was reported to be well tolerated during concurrent chemoradiation in a phase I/II HNSCC study, but its therapeutic benefit remained uncertain and requires confirmation in randomized trials (99). Likewise, a prospective window-of-opportunity study in oral cavity squamous cell carcinoma did not demonstrate a significant improvement in hypoxia-associated gene expression after short-term metformin exposure, underscoring the complexity of translating mitochondrial modulation into measurable clinical benefit (97).

**TABLE 2:** Clinical-stage mitochondrial-targeted therapies with direct or indirect relevance to HNSCC/OSCC.

Drug	Mechanism	Trial (NCT)	Population	Phase	Notes
Metformin	Complex I inhibition/	NCT02325401	Locally advanced	Phase I/II	Early HNSCC studies suggest feasibility and metabolic modulation during chemoradiation; efficacy remains unproven.
	AMPK activation	NCT02949700	HNSCC (incl. oral cavity)		
AT-101	Pan-Bcl-2/	NCT00401349	HNSCC	Phase II	Completed early study with modest activity; mainly supports future combination strategies.
(Gossypol)	LDHA inhibition				
Dichloroacetate	PDK inhibition	NCT01163487	Recurrent head and	Phase I	Head-and-neck-specific metabolic study; feasibility shown, but efficacy remains unproven.
(DCA)			Neck cancer		
Navitoclax	Bcl-2/Bcl-xL	NCT03366103	Recurrent SCLC/	Phase I/II	Non-HNSCC combination trial; included only as an indirect BH3-mimetic precedent.
(ABT-263)	Inhibition		Other solid tumors		
Devimistat	PDH/KGDH	NCT01832857	Advanced/metastatic	Phase II	Non-HNSCC solid-tumor trial; included only as an indirect metabolic precedent.
(CPI-613)	Inhibition		Solid tumors		

Future progress in this field will depend less on adding further empiric agents and more on matching the right mitochondrial vulnerability to the right patient subset. In this regard, candidate predictive biomarkers should be prioritized, including mtDNA mutational burden or heteroplasmy patterns associated with cisplatin adaptation, as well as metabolic profiling features that reflect rewiring of glycolysis, the tricarboxylic acid cycle, glutamine utilization, and redox homeostasis in HNSCC/OSCC (119, 120). Rather than broadly extrapolating from solid-tumor “basket” studies, the next generation of trials should therefore be designed as OSCC-relevant, biomarker-enriched studies that integrate pharmacodynamic readouts, resistance monitoring, and rational combination strategies targeting apoptosis, metabolism, and oxidative stress (121).

At the same time, several translational barriers remain unresolved, including tumor-selective delivery, adaptive metabolic rewiring, redox compensation, and potential on-target toxicity in normal tissues with high mitochondrial demand (122, 123). Addressing these issues will require tighter integration of mechanistic biology, translational pharmacology, and clinically annotated biospecimen studies (124, 125). With such a framework, mitochondrial therapeutics may evolve from an intriguing conceptual strategy into a more precise and testable treatment avenue for OSCC (126–130).
